# Learning does not just happen: establishing learning principles for tools to translate resilience into practice, based on a participatory approach

**DOI:** 10.1186/s12913-023-09653-8

**Published:** 2023-06-16

**Authors:** Cecilie Haraldseid-Driftland, Hilda Bø Lyng, Veslemøy Guise, Hilde Valen Waehle, Lene Schibevaag, Eline Ree, Birte Fagerdal, Ruth Baxter, Louise A. Ellis, Jeffrey Braithwaite, Siri Wiig

**Affiliations:** 1 Centre Faculty of Health Sciences, SHARE - Centre for Resilience in Healthcare, University of Stavanger, Stavanger, Norway; 2 Section for Patient Safety, Dept. of Research and Development, Haukeland University Hospital, Bergen, Norway; 3 School of Psychology, University of Leeds and the Yorkshire Quality and Safety Research group, Leeds, England; 4 Centre for Healthcare Resilience and Implementation Science, Australian Institute of Health Innovation, Macquarie University, Sydney, Australia

**Keywords:** Resilience in healthcare, Learning, Tools, Healthcare systems

## Abstract

**Background:**

Theories of learning are of clear importance to resilience in healthcare since the ability to successfully adapt and improve patient care is closely linked to the ability to understand what happens and why. Learning from both positive and negative events is crucial. While several tools and approaches for learning from adverse events have been developed, tools for learning from successful events are scarce. Theoretical anchoring, understanding of learning mechanisms, and establishing foundational principles for learning in resilience are pivotal strategies when designing interventions to develop or strengthen resilient performance. The resilient healthcare literature has called for resilience interventions, and new tools to translate resilience into practice have emerged but without necessarily stipulating foundational learning principles. Unless learning principles are anchored in the literature and based on research evidence, successful innovation in the field is unlikely to occur. The aim of this paper is to explore: What are key learning principles for developing learning tools to help translate resilience into practice?

**Methods:**

This paper reports on a two-phased mixed methods study which took place over a 3-year period. A range of data collection and development activities were conducted including a participatory approach which involved iterative workshops with multiple stakeholders in the Norwegian healthcare system.

**Results:**

In total, eight learning principles were generated which can be used to help develop learning tools to translate resilience into practice. The principles are grounded in stakeholder needs and experiences and in the literature. The principles are divided into three groups: collaborative, practical, and content elements.

**Conclusions:**

The establishment of eight learning principles that aim to help develop tools to translate resilience into practice. In turn, this may support the adoption of collaborative learning approaches and the establishment of reflexive spaces which acknowledge system complexity across contexts. They demonstrate easy usability and relevance to practice.

## Introduction

Learning is fundamental to quality and safety improvement efforts in healthcare and has been an integral part of researchers’ and policy makers’ agendas for decades. The traditional logic, both within healthcare, workplaces, and education is that learning from adverse events helps improve structures and systems, and avoids future reoccurrences, thereby ensuring safer and better outcomes [1–5]. Within healthcare, this traditional approach - focusing on adverse events and ‘find and fix’ solutions - is known as ‘Safety-I’ [6]. However, recent studies show that despite a range of efforts over the past two decades, the rate of healthcare related adverse events holds steady at between 5 and 10% for hospitalized patients [7–9] and has even been reported to be as high as 24% [10]. This consistency of these figures over time could imply that traditional ‘Safety-I’ methods, such as root cause analysis [11] and checklists, are inadequate for maintaining high quality and safe care [12]. Research within the educational sector has also pointed out the difficulties of learning from error, due to the multi-facetted and complex contexts within which errors occur, which could even imply that this approach is counter-productive [13, 14]. Research within the healthcare setting has therefore called for a radical change in the approach to understanding and improving the quality and safety of patient care. This new theoretical approach, known as Resilient Healthcare or Safety-II, takes into account the complexity of care process and tries to understand and learn from what predict positive outcomes in addition to studying errors [12, 15].

In recent years, interest in resilient healthcare, and in particular, ‘Safety-II’, has increased. The focus here is on everyday work and performance variability. This approach asks: how are patients kept safe in complex, challenging, pressurized environments, through normal working conditions and practices? Understanding how safe care is *created*, and how things go right so often, is seen as a key source of learning [12, 16–21]. Resilient healthcare research explores how healthcare organizations, their staff, patients, and informal carers anticipate, monitor, respond and learn when facing disruptions and/or possibilities for innovation [16, 18, 22, 23]. In this research field, resilience is defined as the capacity to adapt to challenges and changes at different system levels in order to maintain high quality care [6]. Resilient healthcare offers a systems perspective on how individuals, teams, and organizations successfully adapt to their changing circumstances [24]. This systems approach is important since it shifts the responsibility for providing high quality patient care from individuals alone and instead puts the focus on the system’s ability to enable resilient performance among the actors in the system. Learning is central to developing resilient systems– it enables us to develop understanding over time, and to deeply appreciate what happens and why [25, 26].

Based on the premise that resilience in healthcare is a systems perspective, the learning component within resilience refers to organizational learning. This occurs when an organization adapts its enterprise by assimilating new knowledge, while simultaneously exploiting existing knowledge to change and improve their systems, routines, rules and procedures [27]. Given the complexity of healthcare, resilient performance depends on high levels of collaboration and interconnection across different system levels (individuals, teams and organizations), and between different stakeholders (including healthcare professionals, patents, and families) [28–30]. The learning element within the resilient healthcare literature therefore builds on the importance of collaborative learning - that is learning through work and learning together [31, 32]. Learning in professional environments works best when it occurs continuously and is a collective enterprise – when healthcare professionals, patients and families, leaders, and policy makers exchange information, share knowledge, offer support to each other, and coordinate, negotiate and align efforts to deliver care safely [30]. However, beyond these general statements, we lack detailed empirical knowledge about how learning processes for translating resilience to practice occur, how learning principles may support resilience activities, and more specifically how such theoretical positioning can be translated from theory into practice to improve quality and safety of care [6, 12, 30, 33]. In short, we need a more detailed picture of learning processes for resilience in healthcare in situ.

Optimally, complex interventions need an underpinning program theory which describes the mechanisms and contexts that are hypothesized to produce the desired outcomes [34]. To scale up efforts to strengthen resilient healthcare, exploration of the collaborative learning mechanisms that underpin the adaptations, trade-offs, and improvisations that occur when people respond to disruptions is needed [6, 19, 30]. We propose that theoretical anchoring, understanding of learning mechanisms, and establishing foundational principles for learning in resilience are key requirements when designing interventions aimed at strengthening resilient performance. Experts in resilient healthcare argue that learning from both positive and negative events is important, and while many tools and approaches for learning from adverse events have been developed, tools for learning from successful events are limited [35]. The resilient healthcare literature has called for resilience interventions [20, 36], and new tools to translate resilience into practice have started to emerge e.g. [37–40]. In this context, a learning tool can be understood as an artefact that people collectively interact with to support organizational learning i.e. a change in organizational knowledge [41]. In terms of translating theory into practice, the learning tool must add to or transform the situated organizational knowledge [41]. Learning principles, conceptualised as pedagogical ideas, are foundational for any learning tool, process, or activity aiming to translate research or theory into practice [34]. However, to date there is no consensus or evidence around what these learning principles should be. Without this, success and innovation in the field is unlikely to occur.

### The resilience in healthcare program

The longitudinal research program *Resilience in Healthcare* (RiH) (2018–2024) [6, 12] builds on the ideas of adaptive capacity, learning from what usually goes right, and understanding everyday work practices within complex healthcare systems. Its focus is on how people learn collaboratively, in the real world of practice, as a fundamental aspect of resilient healthcare. The project develops resilient healthcare theory and aims to translate resilience capacities into practice through development of a collaborative learning framework and tools [12, 19, 22, 42]. In doing so, the project seeks to advance current thinking in resilient healthcare and help reduce the gap between theory and practice [20, 21, 23, 36].

### Aim and research question

The specific aim of this paper is to describe how the RiH program uses multiple methods and a participatory approach to establish learning principles for tools to help translate resilience into practice. To develop a resilience learning tool for healthcare professionals working in different contexts and across different levels in the healthcare system, the RiH project requires basic foundational principles to ensure and promote translation, relevance, and uptake of any future resilience interventions aimed at testing the tool. The research question guiding our study was: What are key learning principles for developing learning tools to help translate resilience into practice?

## Methods

### Design

We conducted a two-phased mixed methods study [43]. In the first phase the relationships between resilience and collaborative learning were explored and in the second, a set of principles to underpin any collaborative learning tool to translate resilience into practice was developed [12, 19].

### Data collection

Data collection took place over a three-year period and applied a range of data collection and development activities including a participatory approach involving iterative workshops with multiple stakeholders in the Norwegian healthcare system. Table 1 outlines each method of data collection and its purpose. We report data that were collected across the entire process and used in an iterative way to generate the foundational principles for the learning tool.

**Table 1:** Overview of activities, purposes, participants and settings relevant to the development of the learning principles

Type A activities	Purpose	Participants/ Material/numbers	Setting
1.Narrative analyses	RiH theory development and description of the role of collaborative learning within RiH	Narratives from 14 different research projects (70 pages)	Emergency care Primary care Hospital care
2.Literature review	Identify current tools used for translating RiH into practice	Six research papers	Hospital Simulation Government
3.Individual interviews	Exploring adaptive capacities, stakeholder involvement and collaborative learning in relation to resilience	16 interviews with researchers within learning, patient safety, technology, involvement, teamwork, regulation, leadership etc.	Emergency care Primary care Hospital care Transitional care
4.Researcher and designer workshops	Exploring what a learning tool could look like and what it must contain	Two different workshops with a total of 19 researchers within areas such as patient safety, technology, involvement, teamwork, learning, design and programming	Emergency care Primary care Hospital care Design
5.Observations	Identifying relevant learning situation during everyday practice	Structured-, hybrid-, responsive- and coordinating teams (nurses and physicians) 115 h	Hospital
6.Design team meetings	Exploring what a learning tool could look like and what it must contain	40 meetings usually with 2–4 representative from various disciplines such as web- illustrations- and graphic designers, programmers, film-, podcast- and video producers and photographers	Design
7.Involvement panel workshop	Exploring the needs of different stakeholders in different settings and levels	One workshop with 22 participants. Representatives from; patient and family groups, municipality, ombudsman, policy makers, researchers and design team	Hospital Community care Regional and national government
8.Focus group interviews	Feedback on prototype versions of different elements of the learning tool	5 interviews with a total of 17 participants such as healthcare personnel, nurses, leaders, quality managers	Nursing home Home healthcare, Hospital
9.Healthcare personnel workshop	Feedback on prototype versions of different elements of the learning tool	1 workshop with 6 participants including nurses, leaders and nurse assistants	Home healthcare service

In line with our study protocol [12, 19] data collection in the first explorative phase included a scoping literature review of collaborative learning tools in resilience and their pedagogical approach to translate resilience into practice [33]. Data was then collected by interviewing resilience researchers [44] and by reviewing current and completed research projects. A meta synthesis of these data was conducted to understand the research landscape of collaborative learning processes in relation to resilient healthcare [30]. Finding from the explorative first phase analysis informed the following second phase.

The second phase involved several workshops with multiple stakeholders including researchers, practitioners, IT designers, and Norwegian representatives from patient and family groups, municipalities, and policy makers. The workshops provided the RiH project group with feedback on diverse stakeholder views, for example, what a learning tool should look like, what it must address, and how it could be made easily accessible and relevant to the field. This information was fed back to the project group and further refinements were made to develop the learning principles which a collaborative learning tool was to be based upon in a later stage. After these refinements, a synthesis was presented to healthcare leaders and professionals as the tool’s target group. In these workshops with the intended users, participants were asked what they thought an intervention to implement the tool should look like, and what the criteria for successful implementation might be.

### Data analysis

**Fig. 1:**
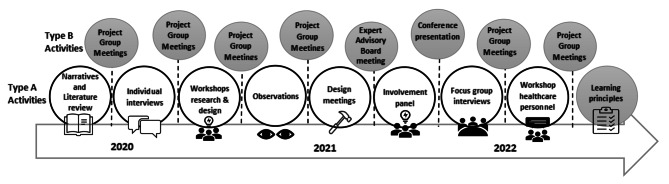
Illustration of the activities in the development process of the learning principles

The analysis to establish the learning principles was a continuous process where members of the project group iteratively integrated input from different stakeholders. The cyclic and iterative process entailed two different types of activity referred to in the following as Type A and Type B activities. Type A activities included data that were collected through the methods outlined in Table 1. These data were then discussed and refined to generate the learning principles in Type B activities which included project group meetings, an expert advisory board meeting, and a conference contribution. The analysis was led by CHD who based on the outcome from the Type A activities drafted and presented various versions of the principles, to all the participants in the different Type B activities. The process lasted through the period from December 2020 to December 2022. Several of the authors contributed at various stages, while CHD, SW, ER, VG, HBL, BF, LS, and HVW contributed in all Type B activities such as the project group meetings. Please see Fig. 1 for overview of the process and the different activities included.

The analysis was inspired by joint display of data [43] where different data sources contributed to establishing the learning principles. A joint display of data gives the researcher an opportunity to combine different datasets and display them in an integrated manner to see what overlaps [43]. All principles were refined and discussed throughout the entire process and across all of the Type B activities. However, the type A activities contributed with general input from the stakeholders, related to what is important when designing a learning tool, while the Type B activities was used to refine and discuss how the input form the stakeholder could be operationalized through the different principles. How each of the learning activities feed into the different principles is displayed in Table 2. Participants in the Type B activities had extensive knowledge of the resilience in healthcare literature, which more specifically build on the collaborative learning values [31, 45] and organizational learning theory [27, 46] such as described in the introduction. The derived principles are therefore grounded in a combination of different stakeholders’ needs and experiences, and in the literature.

**Table 2:** Overview of how type A activities contributed to the learning principles

Principle	Contributing Type A activity
**Principle 1: Use a collaborative approach**	1, 2 & 4.
**Principle 2: Create collaboration across levels stakeholders and contexts**	1, 2, 3, 4, 5, 6, 7, 8 & 9.
**Principle 3: High flexibility that accommodate time and place**	3, 4, 7 & 8
**Principle 4: Ensure usability and easy access**	3,4, 6,7 & 8
**Principle 5: Highly relevant for context**	1, 2, 3, 4, 5, 6, 7, 8 & 9
**Principle 6: Create space for reflection**	2, 4, 8 & 9
**Principle 7: Create awareness of adaptive capacities**	3, 4, 5, 7, 8 & 9
**Principle 8: Share examples of good practice**	3, 4, 7, 8 & 9

## Results

**Fig. 2:**
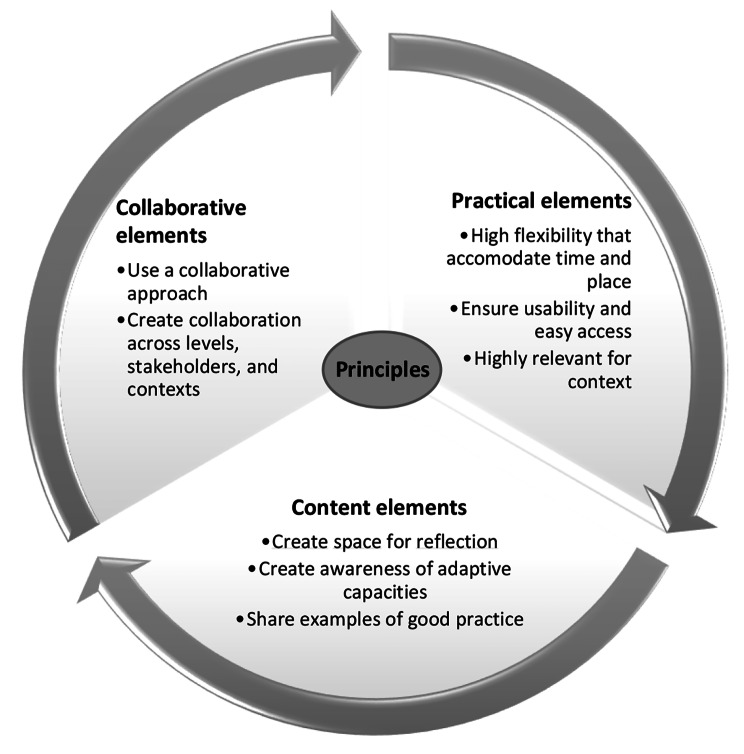
Overview of learning principles for translating resilience into healthcare practice

We present the eight key principles for developing learning tools to help translate resilience into practice, and the rationale for these. The principles consist of collaborative elements, practical elements, and content elements. In Fig. 2, we visualize the principles and how they form the foundation of the tool.

### Collaborative elements

#### Principle 1: use a collaborative approach

This principle mainly stems from workshops between researchers and designers and the literature review. The researchers stressed their previous experience with similar projects which demonstrated the utility of using a collaborative approach - people working and learning together through sharing experiences. This principle was strongly linked to principle two, ‘*Create collaboration across levels, stakeholders and contexts’*. The literature review findings [35] showed that several existing tools for translating resilience into practice reported difficulties in understanding the organizational aspects of resilience, attributed mainly to the fact that past tools employed an individual approach rather than a system perspective.

#### Principle 2: create collaboration across levels, stakeholders, and contexts

This principle became evident across the workshops and the literature review, narratives, focus group interviews and observations. It reflects the fact that resilience is expressed at multiple levels of the system ranging across units, departments, and organizations. During the Type A activities, a range of meeting places were created where participants from different levels or settings could interact and exchange information. This was found to enrich their perspectives. Through the observations in hospitals, such enrichment was also seen in practice, but time and resources were often rate-limiting factors. In the workshops and interviews, further examples of activities which had created valuable exchanges of information and viewpoints were used to exemplify rich learning occasions that created an opportunity to understand a situation, case or problem from different angles.

### Practical elements

#### Principle 3: high flexibility, that accommodate time and place

This principle was primarily derived from the focus groups with healthcare personnel and the involvement panel, but it was also recognised by the design team and the researchers, and through the observations and the literature review. This principle was primarily related to the busy environment facing most in situ healthcare personnel, but also relates to principle two of creating space for collaboration across stakeholders, levels and contexts. Having the time and places to engage in such collaborations were reported as very real challenges within the healthcare system. Any resilience learning tool therefore needed to be designed with high levels of flexibility in mind. Flexibility also refers to the need to facilitate the involvement of a variety of different participants that reflect the stakeholders and characteristics of the different teams, units, and organizations for which the tool is being designed.

#### Principle 4: ensure usability and easy access

This principle was highlighted through the healthcare, involvement panel, researcher and designer-workshops, all of which agreed that easy access is one of the most important elements of a successful tool. Several participants had been part of previous projects where poor accessibility and user friendliness had hindered the use of an intervention or tool, due to either technological or distributional issues. Easy access to the tool is one of the most pressing underpinning principles, and failure to design an accessible and intuitive product will undermine its ultimate value. Challenging working environments with limited available time makes user friendliness even more important since staff have no time to spare in figuring out how to use a tool. This principle was also linked to principles one and two. Participating healthcare personnel also stressed that they already had multiple different technologies and tools which required various passwords and usernames, and that yet another log in code was not welcome. On the other hand, participants across healthcare settings stressed that technology-based tools had become more commonplace during the COVID-19 pandemic and so would now be more acceptable and more likely to be accessed than before.

#### Principle 5: highly relevant for context

Relevance for context was one of the most frequently mentioned principles by all the different stakeholders. Together with principle three, ‘high flexibility that accommodate time and place’ and principle four ‘Ensure usability and easy access’, this was the principle with the highest level of agreement. All healthcare personnel and organisations are continuously presented with new ideas, systems, procedures, tools, technology, equipment, and processes. When there is a high demand for care delivery but few resources available, time is precious. What to spend time and resources on must be carefully considered. Relevance thus becomes a highly rated criterion for users. The user must find the tool worth their while and value-adding, and they must regard it as relevant to their context. Relevance does not necessarily mean a high level of authenticity or an exact replication of the intended users’ specific context, but rather the opportunity to customize the use and content of a tool to their own contextual needs.

### Content elements

#### Principle 6: create space for reflection

The sixth principle, ‘create space for reflection’ stemmed mostly from the individual interviews and the researcher and designer workshops. Several participants advocated using reflection as a pedagogical approach, particularly in combination with principle two. Participation in previous research projects had shown the research team the value of giving different stakeholders the opportunity to vocalize thoughts and opinions in groups. Such practices had provided participants with both the opportunity to understand a situation from a different perspective, as well as the chance to learn from others. In a hectic healthcare environment, opportunities for sustained reflection rarely present themselves, creating the need for a structure that helps generate such spaces. Reflexive spaces were considered fundamental for leveraging greater levels of resilience in practice. Through both workshops and the literature review, we identified not only a need to make room for reflections, but also a need to facilitate the content of reflections. While the workshops uncovered an uncertainty about how to facilitate good reflections, the literature review showed the need for linking this principle with principle seven ‘create awareness of adaptive capacities’. This entailed making room for guided reflections which help participants both understand the organizational aspects of resilience and increase their awareness of what adaptive capacities are, while simultaneously providing a structure of what to reflect upon to prevent ‘unproductive’ reflections.

#### Principle 7: create awareness of adaptive capacities

As resilient healthcare is likely to be a new approach to most potential users of a resilience learning tool, the need to create awareness of what adaptive capacity is and what it entails was widely emphasised across individual interviews, focus group interviews, and all workshops. Researchers and participating healthcare professionals agreed that the concepts of resilience and adaptive capacity were understandable and easily recognizable if they were clearly explained and illustrated though examples, figures or explanations. However, it could be challenging to identify adaptive capacities in real-world practice and to understand how adaptations influence and affect other adaptations. A tool therefore needs to help users understand and identify adaptive capacities within their respective settings.

#### Principle 8: share example of good practice

Across the workshops as well as the interviews, participants indicated that a good tool would provide opportunities for people to learn from others and to be inspired and motivated through examples of good practice. This principle is closely linked to principles one and two, where participants’ previous experiences had showed them the importance and value of collaborating, sharing, and understanding behaviours, processes, and procedures from different perspectives. This principle also relates to the need to understand what ‘good’ practice is and what it entails and to encourage discussions about what is ‘good enough’. Sharing examples of practice is an important way of illustrating that good practice does not necessarily mean something that is extraordinary, but rather that it is often ‘ordinary’ everyday work that is regarded as good practice. Several of the discussions revealed that participants wanted examples and ways of sharing good practice.

## Discussion

The resilient healthcare literature has called for tools and interventions to translate resilience into practice [12, 20, 21, 30]. This project is one specific response to those calls. Through a participatory and mixed methods approach, we set out to ground a resilience learning tool in the real life needs and expectations of a diverse group of stakeholders, alongside evidence from the literature.

### Learning principles and program theory to translate resilience into practice

Knowledge translation and improvement programs in healthcare need to define their program theory as to how interventions are anticipated to work. Our study provides a starting point for developing a program theory to support the development of a collaborative learning tool to help translate resilient healthcare into practice. Our interest in healthcare as a complex adaptive system [47] implies that when we intentionally try to translate theoretical concepts, such as resilience, into a practical learning tool, we need to integrate the understanding of this complexity. Healthcare is a multi-layered system; there is perennial emergence, and, oftentimes, blurred boundaries [30, 31]. The elements of collaboration, content that recognizes complexity, and practical usability are pivotal learning principles that should be linked to an appropriate program theory.

Learning from everyday work and success is fundamental to resilient healthcare [16, 18, 48]. Our learning principles build on these ideas and advance them by taking everyday practices into account in the reflexive spaces [49]. Our approach is not just a quick fix, but reinforces the need for structures, principles, and support systems to nurture learning. Grounding our learning principles in real life needs and relevant theories helps strengthen the potential for subsequent tools and interventions to succeed. The end result is relevant and targeted resilience learning tools, and effective intervention strategies.

Our work indicates strong support for Hollnagel’s [48] four resilience potentials (anticipate, monitor, respond, learn) and in particular the importance of the potential of learning. However, while the importance of learning is clearly pointed out within the resilience in healthcare literature [26, 48], the role of learning, how to go about this process of learning, how tools can be developed to translate resilience into practice, and how resilient performance can be supported through learning is scarcely described [12, 19, 49]. The principles provided in this paper are therefore outlined as broader and more general guidelines pointing out the importance of design and relevance. While it could be argued that such issues have been addressed within other relevant literature such as the e-learning literature [50, 51], several of the existing resilience learning tools report issues related to their implementation process. In particular, they highlight the difficulties related to getting the participants to engage with the tools, grasp the concept of resilience, and operationalize the complexity of the concept [37–39]. Given the novelty of the resilience in healthcare literature and the lack of description relating to how and what to learn, this indicates that there is a need for general or more abstract principles, that in the future could be developed and refined into more specific principles.

### Integrating a wider range of learning opportunities

Learning from everyday success and how clinical work routinely keeps patients safe are two pillars of resilient healthcare. Resilient healthcare is an antidote to excessively focusing on things that go wrong, which has been a preoccupation of safety commentators and practitioners for too long. Our work enables new ways of thinking about learning, and tries to anchor work practice improvement within a carefully-crafted understanding of the conditions under which people work [16, 48]. Learning from what goes wrong *and* what goes well in everyday work are complementary perspectives, providing different learning opportunities. In the traditional Safety-I paradigm, people try to figure out what contributes to negative outcomes [16]. Such methods are considered reductionistic and linear [18], yet assumed to contribute to learning. Learning from everyday work and its successes is different [39] and requires much greater attention than it currently receives [27]. But learning from what goes well also needs facilitation and guidance, as this approach is in its infancy.

Yet it is always wise to remember that this will not be easy. Healthcare is rightfully described as a complex adaptive system where performance is not linear, and where situations cannot be broken down into single elements [52].

## Strengths and limitations

The development of the learning principles in this study was a result of an iterative approach of discussions and refinement, featuring a range of activities with different stakeholders. While the range of activities included in this study ensured that multiple different views were taken into consideration, the stakeholders involved in the Type A activities were only from a Norwegian context. The specific healthcare context, including fewer private hospitals and a government funded healthcare system, could therefore have influenced the findings. On the other hand, the Type B activities included international participants who thereby contributed with important international context during the refinement of the principles. However, the study could have benefitted from a broader international collaboration where stakeholders from different context contributed in all activities.

The learning principles were developed by a group of researchers who acted concertedly and gathered information from a wide range of sources which contributes positively to the trustworthiness of the findings. However, involving such a wide range of parties could risk fragmentation and difficulty in seeing the holistic picture. This issue was prevented by having one researcher (CHD) leading the developmental process and through dividing the process up into multiple stages, where consensus was reached between each stage. The approach could have been even more directly participatory, by asking the stakeholders to help develop and form the principles or contribute as a part of the research team. It could be viewed as a limitation that this study was not grounded within a theoretical learning framework, since the study touches upon educational and learning components. However, the aim of this study was to elaborate on the learning element within resilience in healthcare theory, and to facilitate translation of resilience into practice. The study was therefore grounded within the resilience in healthcare theory so as to best suit the study’s purpose. Future studies might benefit from investigating the connections between the learning element in resilience in healthcare theory and how this relates to and builds upon different general learning theories.

Future research should test the principles in a broader international context and further describe the importance of learning within the resilient healthcare literature to improve the ability to operationalize the resilience in healthcare theory through further developing the principles.

## Conclusion and implications

We established eight principles for learning tools that aim to support the translation of resilience into practice. The principles provide a sound foundation for use and uptake of research-based knowledge in resilient healthcare. Understanding the view of diverse stakeholders in healthcare, alongside the incorporation of input from researchers and findings from previous research, enables an integrative and participatory foundation for translating resilience into practice in a way that has not been demonstrated in the research literature previously.

We do not yet know the outcomes from testing a tool based on our principles as this research is currently ongoing. How we define success in translating resilience into practice and how we can measure outcomes and evaluate the processes involved, remains an under-researched area. Knowledge and understanding of what types of resilience tools work for whom and in what contexts is not yet clear and requires more research.

## Data Availability

The datasets used and analysed during the current study are available from the corresponding author on reasonable request.

## List of abbreviations

RiH: Resilience in Healthcare
